# Effects of confining pressure and loading direction on the mechanical behavior of schist with high content and aggregation degree of mica

**DOI:** 10.1371/journal.pone.0344580

**Published:** 2026-03-20

**Authors:** Qiliang Liu, Jing Xiang, Xiaomeng Yin, Kun Song

**Affiliations:** 1 School of Civil Engineering, Science and Technology College of Hubei University of Arts and Science, Xiangyang, China; 2 School of Civil Engineering and Architecture, Hubei University of Arts and Science, Xiangyang, China; 3 Hubei Key Laboratory of Vehicle-Infrastructure Collaboration and Traffic Control (Hubei University of Arts and Science), Xiangyang, China; 4 Hubei Key Laboratory of Disaster Prevention and Mitigation, China Three Gorges University, Yichang, China; China Construction Fourth Engineering Division Corp. Ltd, CHINA

## Abstract

This article focuses on the mechanical anisotropy of the schist with high content and aggregation degree of mica, which are prone to causing local collapse, large deformation, and support failure in tunnel engineering. The fabric characteristics of rock and the response of mechanical properties to confining pressure and loading direction was emphatically explored by micro tests and compression tests with confining pressures ranging from 0 to 20 MPa. Micro tests reveal that the flaky micas are highly aggregated and directionally arranged in the “matrix” composed of granular minerals and directional cracks are developed along the cleavage or the boundaries of mica. Mechanical tests indicate that the peak strength and elastic modulus of schist subjected to uniaxial compression vary in a shoulder form and U-shaped form with the orientation angle *α*, respectively. The confining pressure *σ*_3_ nonlinearly weakens the anisotropy of schist and the transition of rock behavior from anisotropic to isotropic occurs at a critical confining pressure. Moreover, the critical ratio has a negative correlation with the most representative uniaxial compressive strength and strength anisotropy degree under uniaxial compression. As *σ*_3_ increases, the failure mode changes from tension and shear composite to pure shear at *α* = 90° and from splitting and shear composite to plastic kinking at *α* = 0°, while maintains sliding shear at *α* = 30°. However, under the low *σ*_3_, the tested schist is different from the schist with low content and aggregation degree of mica in the failure mode at *α* = 0° and 30°, which is attributed to the differences in the microstructures of two schist. These findings yield an in-depth understanding of the mechanical behavior of foliated rocks and offer important references for the stability evaluation and support design of tunnel surrounding rock.

## 1 Introduction

Many rocks in nature unavoidably produce inherent weak planes during the process of diagenesis, including flow banding, bedding, schistosity, gneissosity, etc. Significant differences in mechanical properties are revealed in these rocks encountering loads in different directions. In various types of geotechnical engineering, mechanical anisotropy often plays an important role in controlling the stability of geotechnical bodies [1]. For example, in slope engineering, the spatial location and failure mechanism of dangerous parts of the slope vary with the attitude of weak planes [2], and the stability of the slope also changes accordingly [3]. In underground engineering, the failure modes of the potential risk points in the surrounding rock are closely related to the angle between the maximum principal stress and the weak planes [4,5]. A good understanding of the mechanical anisotropy of geotechnical materials is of great significance for the design department in choosing the correct support method and determining the reasonable support parameters.

Except for some igneous rocks and sedimentary rocks, a considerable proportion of metamorphic rocks such as schist, slate, gneiss, etc. exhibit significant anisotropy [6]. The mechanical properties of these foliated rocks have been investigated by numerous scholars based on laboratory experiments. Most of the previous studies focus on the compressive strength and deformation properties. Some common conclusions have been recognized as follows.

a) The compressive strength as a function of loading direction is generally characterized by U-shaped, shoulder-shaped and wave-shaped variations [7,8]. The maximum compressive strength is at *α* = 0° or *α* = 90° [9] (*α* represents the orientation angle between the loading direction and weak planes). The angle *α*_m_ corresponding to the minimum compressive strength varies with the rock type, and Nasseri et al. [10] points out that the *α*_m_ is theoretically equal to 45°-ϕw/2 (ϕw represents the internal friction angle of weak planes).
b) The elastic modulus exhibits a U-shaped or monotonic variation with the loading direction [11,12]. Generally, the elastic modulus of anisotropic rocks in the loading direction parallel to weak planes is greater than that perpendicular to weak planes.
c) The dominant effect of weak planes is gradually weakened by the increasing confining pressure [13]. It is manifested by the weakening of the orientation dependence of the mechanical indexes and the decrease of the anisotropy degree defined by the ratio of maximum to minimum compressive strengths with the increase of the confining pressure [14,15]. In addition, the *α*_m_ corresponding to the minimum compressive strength tends to increase in response to the increasing confining pressure [10,16,17].

In terms of mineral composition, foliated rocks are characterized by the presence of phyllosilicate minerals such as mica, chlorite, illite, montmorillonite, etc. Previous studies have shown that as weak mineral phases in rocks, the proportion of phyllosilicate minerals greatly controls the strength of rocks [18]. It is recognized that for similar rocks, a higher content of weak mineral phases generally reduces strength but enhances toughness of rocks. In the progressive failure process of brittle rocks, phyllosilicate minerals often play an important role in inducing the initiation of new cracks, promoting crack coalescence and triggering shear localization [19,20]. Shea et al. [21] found that the micromechanics of anisotropic failure and mechanical behavior of foliated rocks are strongly influenced by the abundance, concentration and spatial arrangement of phyllosilicate minerals.

The Wudang Group schist containing obvious schistosity planes was formed in Northwest Hubei, China. During tunnel excavation, a sequence of light-colored rocks mainly composed of quartz mica schist was exposed. In order to provide theoretical guidance for tunnel support design, the mechanical anisotropy of this type of quartz mica schist has been studied by multiple scholars. Although the mineral composition of metamorphic rocks in the same region is highly consistent, the mineral content and micro parameters describing mineral distribution characteristics exhibit spatial variability due to the complex formation environment [8]. The previous research on this type of quartz mica schist is still lacking in comprehensiveness, as most attention has been focused on the uniaxial compressive mechanical behavior of strong brittle schist containing low content and aggregation degree of mica [22–24]. Perhaps due to the low success rate of sample preparation, the triaxial compressive mechanical behavior of Wudang Group schist containing high content and aggregation degree of mica has been rarely explored.

It is worth noting that high content and aggregation degree of mica can significantly enhance the orientation dependence of the mechanical properties of schist, and may also cause significant deformation, shear slip, and progressive failure along the schistosity plane, thereby increasing the risk of local collapse, large deformation, and even support failure in tunnels. Therefore, in-depth research on the anisotropic mechanical behavior of such compressed schist is of great value for understanding the failure mechanism of foliated rocks and guiding the safety design of relevant tunnel engineering.

In this study, micro tests were conducted to analyze the typical microstructure of the mica-rich schist. Uniaxial and triaxial compression tests were performed on cylindrical specimens with different orientation angles to study the mechanical behavior of the schist. Moreover, the response pattern of strength anisotropy and failure modes of the mica-rich schist to the confining pressure were analyzed.

## 2 Composition and microstructure characteristics

**Fig 1:**
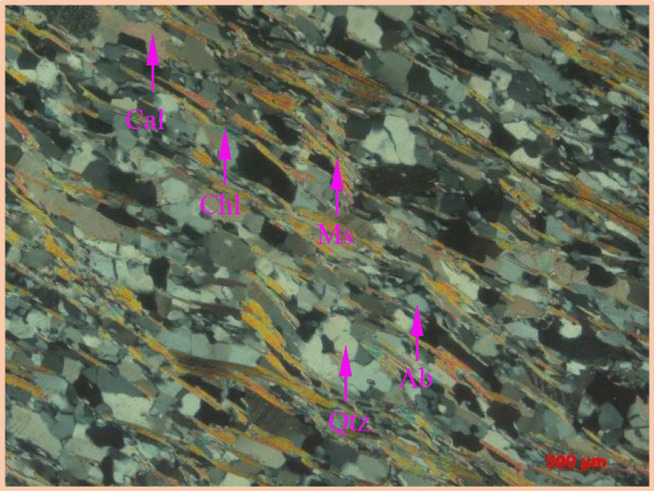
Mineral distribution of the schist sample.

The schist sample was cut into small pieces along the direction perpendicular to the schistosity planes, and a series of operations including gluing, grinding, polishing, etc. was carried out on the pieces to make thin sections. Then, polarizing microscope (PM) was utilized to observe and analyze the composition and distribution characteristics of minerals in the rock. Based on the morphology and optical characteristics of minerals (Fig 1), it can be concluded that the schist is mainly composed of quartz, feldspar, mica, and calcite. Small amounts of chlorite, as a weathering product of mica, were also observed. These minerals were divided into two categories based on their morphology. One is granular minerals, including quartz, feldspar, and calcite, and the other is flaky minerals represented by mica.

Due to the influence of the geological environment, granular minerals exhibit a certain degree of elongation. The Wudang Group schist is located in the South Qinling orogenic belt and has been subjected to multiple tectonic processes. Under the action of deviatoric stress caused by tectonic movement, the granular particles in the rock undergo plastic deformation, accompanied by pressure dissolution at the mineral boundary. Following the theory of Gibbs internal energy, chemical migration occurs from the boundaries subjected to high stress (with high Gibbs internal energy) to that subjected to low stress (with low Gibbs internal energy), leading to a certain degree of particle elongation.

The flaky minerals represented by mica present preferential orientation. During the diagenesis process, fluids stored in the intergranular pores inside the original rock flowed out due to the high stress. Under the combined effect of fluids and stress, the randomly oriented flaky crystals underwent mechanical rotation and tended to be oriented in a certain direction. Unlike the rock containing low aggregation degree of mica, in this type of rock sample, mica minerals are stacked and arranged in a streamline pattern in the “matrix” composed of granular particles, exhibiting highly aggregated characteristics.

**Fig 2:**
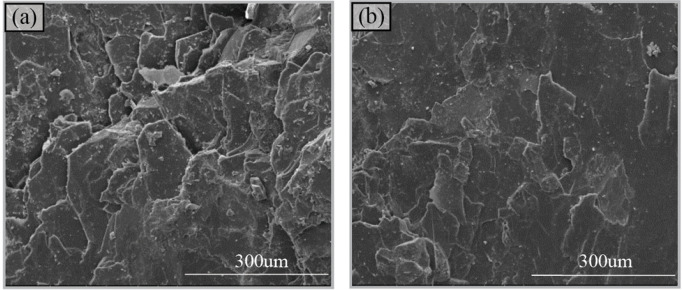
Micro morphology of fracture surface under ESEM, (a) stacked flaky mica and (b) smooth fracture plane.

Environmental scanning electron microscopy (ESEM) was utilized to make morphological observation of sections that were naturally cracked along the schistosity planes by hammering. Fig 2a shows that flaky mica aggregates and stacks together like tiles along the schistosity planes. Under the influence of external force, damage preferentially propagates along the cleavage planes and grain boundaries of flaky minerals, ultimately generating a smooth fracture plane (Fig 2b). Based on the observations of ESEM, it can be inferred that mechanical weak zones exist in areas where flaky minerals aggregate. These zones prevent the effective storage of energy, causing the schist to fracture readily under external loading and release energy along such fracture zones.

**Fig 3:**
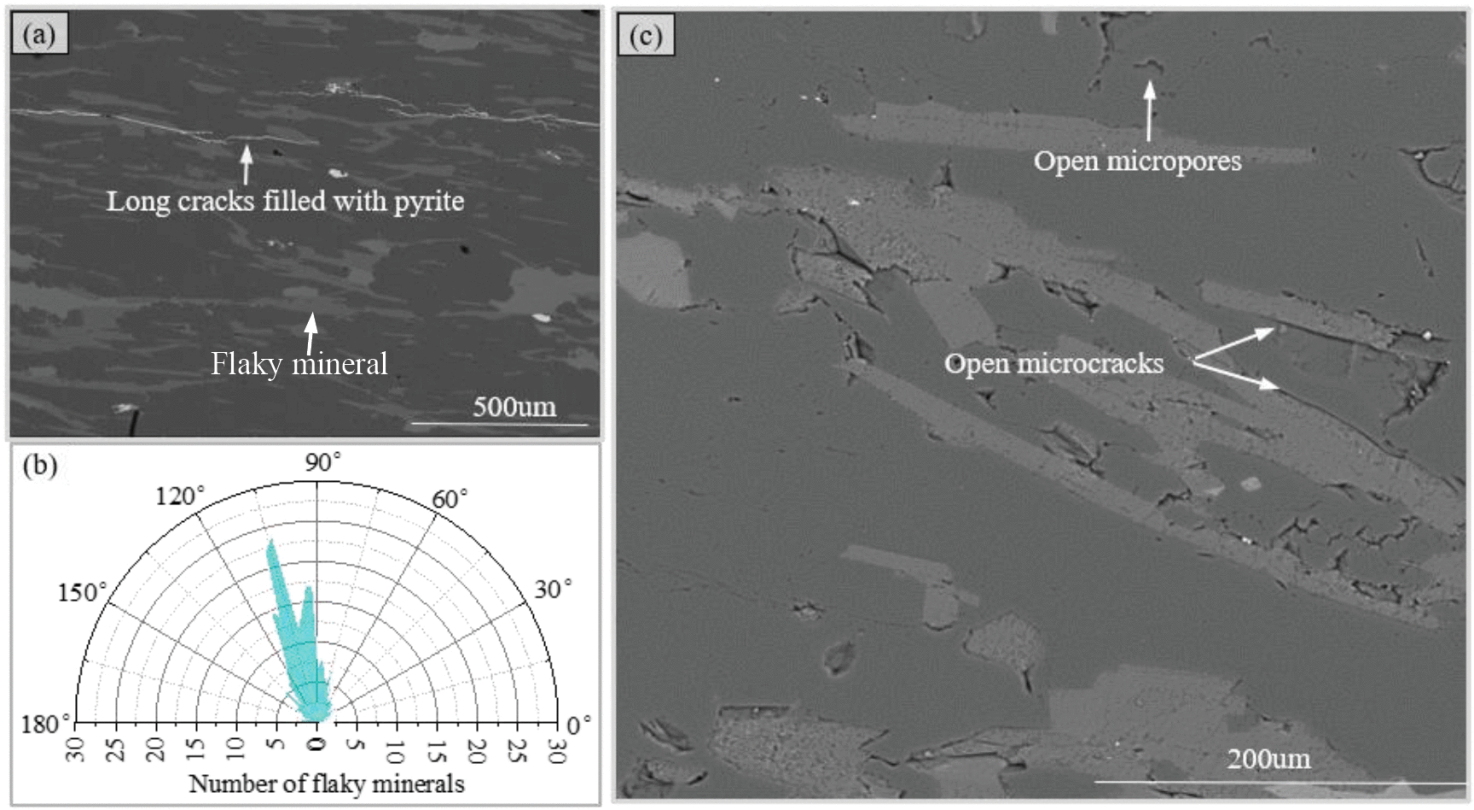
Grayscale images obtained by ESEM and the relevant statistical graph, (a) long cracks in the schist sample, (b) rose diagram of flaky minerals orientation and (c) microcracks and micropores in the schist sample.

Then, ESEM was used to observe optical sections of the rock sample for analyzing its microstructure characteristics. In the grayscale images obtained by ESEM, minerals are distinguished by the difference in brightness and darkness. Constituent elements at some represented points were identified using energy dispersive X-ray spectroscopy (EDS) to verify the analysis results of mineral composition based on the observation of PM. Additionally, trace amounts of pyrite that were overlooked under PM were nevertheless observed under ESEM. The minerals were found to fill in the slender cracks of the rock in the form of veins (Fig 3a), suggesting that the rock was in a reducing environment in the later stage.

The image processing and analysis software Image-Pro Plus (IPP) was utilized to perform statistical analysis on parameters such as the mica area percentage (MAP), the preferred orientation degree (POD) of mica, and the aspect ratio (AR) of mica in grayscale images. The results indicate that the MAP and POD of mica are 29.57% and 0.79, respectively. In the current study, POD is quantified as 1 minus the ratio of the standard deviation to the mean of orientation angle of mineral grains. Here, the orientation angle of mica grains (S1 File) was measured as the angle between the grain’s long axis and the vertical axis of the grayscale image. Statistically, these angles are concentrated between 100° and 110° (Fig 3b). The AR of mica mostly ranges between 2 and 5. A considerable number of mica grains aggregate into clusters, significantly increasing their aspect ratio. Elongated mineral clusters with a high AR (>9) account for approximately 9.36% of the total mica grains.

The high precision of ESEM is conducive to observe the micro defects of rocks, which are considered to be microscopic performance associated with mechanical properties of rocks. According to the state and scale of defects, they can be divided into three categories. The first category is long cracks that have been filled with minerals (Fig 3a). Induced by geological stress after rock formation, the cracks can extend up to 1000 um. The second category is open microcracks with an extension length ranging from 10um to 150um. They are generated by local disturbance stress caused by engineering construction, sampling or sample preparation. The third category is the open micropores that randomly distributed at the particle gaps. The appearance of micropores is likely attributed to the combined effects of chemically active fluids and mechanical disturbances.

Despite differences in the scale and state, the distribution characteristics of the first two types of defects exhibit a high degree of consistency. Both of these defects are distributed in flaky minerals or along the boundaries of flaky minerals (Fig 3a and 3c). On the one hand, a group of extremely complete cleavage developed in the mica minerals constitutes mechanical weak planes. On the other hand, the significant differences in stiffness and toughness between flaky minerals and surrounding granular minerals are apt to cause stress concentration at the contact boundaries [25]. Under external forces, cracks are prone to initiating and propagating at these weak planes and stress concentration zones. Controlled by the spatial arrangement of flaky minerals, both categories of defects exhibit directionality and they are collectively referred to as directional cracks.

## 3 Mechanical properties

Cylindrical specimens (50 mm in diameter and 100 mm in height) with different orientation angles were drilled from the large volume rock blocks taken from the engineering site. Following oven drying at 105°C, the specimens were screened based on measurements of their density and P-wave velocity. First, density and P-wave velocity were averaged for specimens with the same orientation angle. Then, specimens with test values close to the average were retained for mechanical testing. Finally, uniaxial compression tests were conducted on specimens with *α* = 0°, 30°, 45°, 75° and 90°, employing displacement-controlled axial loading at a rate of 0.1 mm/min. Triaxial compression tests were performed on three representative groups of specimens with *α* = 0°, 30° and 90°. Each group of specimens was subjected to the confining pressures of 5, 10, 15 and 20 MPa, respectively. The range of confining pressure was determined based on the burial depth of the tunnels in the project area and the tests were conducted at a constant axial loading rate of 0.1 mm/min.

### 3.1 Uniaxial compression

**Fig 4:**
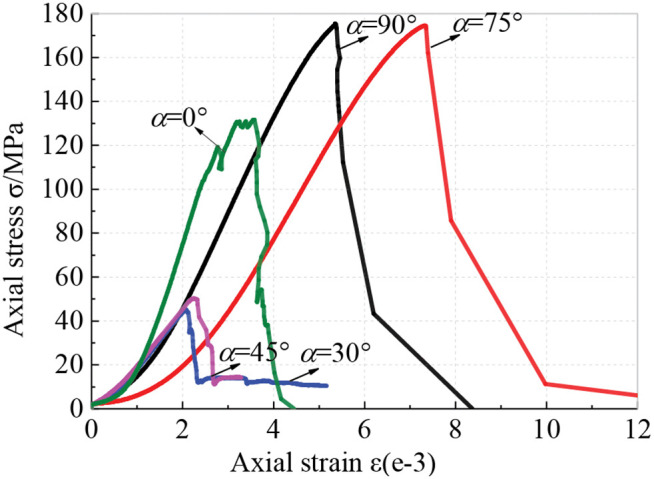
Stress-strain curves of specimens subjected to uniaxial compression.

The stress-strain curves of the specimens subjected to uniaxial compression are shown in Fig 4. All of the curves rapidly fall after reaching their peak points, suggesting the occurrence of brittle failure of the specimens. As the axial strain gradually increases, brittle rocks undergo progressive deformation and damage. According to the characteristics of the stress-strain curves, this progressive process can be divided into five stages.

(1) Compacting stage. This stage is characterized by a concave curve segment. The original open defects in the rock such as micropores and microcracks are rapidly closed under compressive loading. Along with being compacted, the rock produces early irrecoverable nonlinear deformation.
(2) Linear elastic stage. Stress is almost linearly related to strain, and the rock undergoes linear elastic deformation, leading to a rapid accumulation of elastic energy.
(3) Crack stable growth stage. Once the external load reaches a critical value, the initiation of new microcracks occurs within the rock, causing the strain to deviate from linear elasticity. The stress-strain curve shows a slight upward convexity. At this stage, independent microcracks undergo stable propagation, and the microcracks will grow only when the load continues to increase [26].
(4) Crack unsteady growth stage. Once the stress increases to the yield point, microcracks in the specimen rapidly propagate and coalesce. At this stage, the most significant structural changes occur owing to the rapid accumulation of internal damage [27]. Microcracks continue to grow even if the loading is stopped [28]. Corresponding to this stage, the curve is significantly upwardly convex.
(5) Strain softening stage. After the peak point of the curve, the internal structure of rock is further damaged, and macroscopic fracture planes appear, accompanied by a rapid drop in the bearing capacity of rock.

Although all the stress-strain curves exhibit the common five stages of progressive deformation and damage of rock, there exist some differences in the curve characteristics of specimens with different orientation angles. In the early stages of loading, the curves of specimens with *α* = 90° and 75° has more significant bending concave than those of other specimens, which is closely related to the angle between the axial load and directional cracks. Crack closure is favored when the axial load is applied at a high angle to the directional cracks, due to the greater resolved normal stress across them. In the linear elastic stage, the slopes of the curves of specimens vary with their orientation angles. At *α* = 0°, the curve has the largest slope, suggesting that the schist possess a greater anti-deformation ability when loaded parallel to the schistosity planes. The comparison shows that in the crack growth stages, the curve characteristics of specimens with *α* = 45° and 30° are relatively vague, which is dependent on the specific sliding failure mode along the schistosity planes. In this mode, new microcracks propagate and connect at a faster rate, thereby giving rise to the distinctive character of the stress-strain curve. In addition, the morphology of the post peak curve indicates that, unlike the complete strength loss of other specimens, the specimens with *α* = 45° and 30° retain a certain residual strength. It is found that the curve of specimen with *α* = 0° has high-frequency fluctuations near the peak point, which is attributed to its splitting failure mode characterized by the gradual appearance of multiple fracture planes along the schistosity planes.

In summary, according to the curve characteristics, the schist specimens can be divided into three categories. Specifically, specimens with *α* = 90° and 75° belong to the first category. Their curves exhibit typical characteristics of brittle rocks, with clear division of progressive deformation and damage stages. The second category is represented by the specimen with *α* = 0°, and the curve is characterized by obvious undulating and fluctuations. The third category of specimens such as that with *α* = 45° and 30° has an indistinct crack growth stage and the residual stress phenomena. It is worth noting that the differences in the curve characteristics of specimens arise from orientation-dependent failure modes in the tested schist. The failure modes of the three categories of specimens are axial tensile failure, splitting failure and sliding failure, respectively, which are consistent with previous studies [16,29–31].

**Fig 5:**
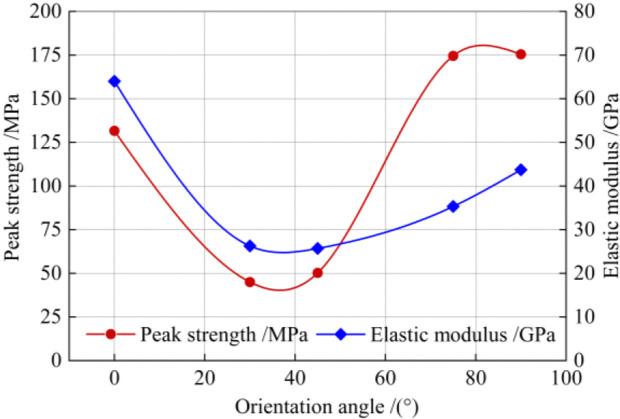
Mechanical indicators of the tested schist subjected to uniaxial compression.

It is well known that the elastic modulus $E_{\text{u}}$ and uniaxial compressive strength (peak strength) $\sigma_{\text{uc}}$ are two important indicators representing the mechanical properties of rocks. In order to analyze the response pattern of mechanical indicators of the tested rock to the loading direction, the variation curves of $E_{\text{u}}$ and $\sigma_{\text{uc}}$ with respect to the orientation angle *α* are plotted in Fig 5. It was found that the $\sigma_{\text{uc}}$ of quartz mica schist varies roughly in a shoulder pattern with the angle *α*, and $\sigma_{\text{uc}}$ at *α* = 90° is greater than that at other angles.

Nasseri et al. [10] conducted uniaxial compression tests on quartz schist, chlorite schist, quartz mica schist, and biotite schist, and analyzed the correlation between $\sigma_{\text{uc}}$ and *α*. Their test results show that the specimen with *α* = 90° has the maximum $\sigma_{\text{uc}}$. Moreover, they found that the specimens with *α* = 90° have the minimum dispersion of strength test values due to relatively uniform stress distribution throughout the planes of anisotropy. Therefore, $\sigma_{\text{uc}}$ at *α* = 90° is generally regarded as the most representative compressive strength $\sigma_{\text{ruc}}$ of anisotropic rocks.

In the present study, the minimum $\sigma_{\text{uc}}$ of quartz mica schist was found at *α* = 30°. This finding aligns with prior work indicating that *α* corresponding to the minimum $\sigma_{\text{uc}}$ lies within the range of 30°–45° [6]. The U-shaped elastic modulus curve is clearly displayed for the tested schist, with the maximum $E_{\text{u}}$ at *α* = 0° and the minimum value at *α* = 45°. A similar trend was observed for foliated rocks tested by Cho et al. [29] and Kim et al. [32]. Based on the test results of uniaxial compression, the compressive strength of the tested schist first decreases and then increases with increasing orientation angle. The angles *α* = 0°, 30°, and 90° well serve as representative orientations for quantifying the strength anisotropy of the schist. Furthermore, previous studies indicate that the failure mode of foliated rocks varies significantly with the loading direction, exhibiting distinct characteristics at large (typically 60°–90°), medium (typically 15°–60°), and small (typically 0°–15°) orientation angles [17,29,32]. Therefore, the specimens with three representative angles of 0°, 30° and 90° were selected to carry out the triaxial compression tests.

### 3.2 Triaxial compression

#### 3.2.1 Stress-strain curves.

**Fig 6:**
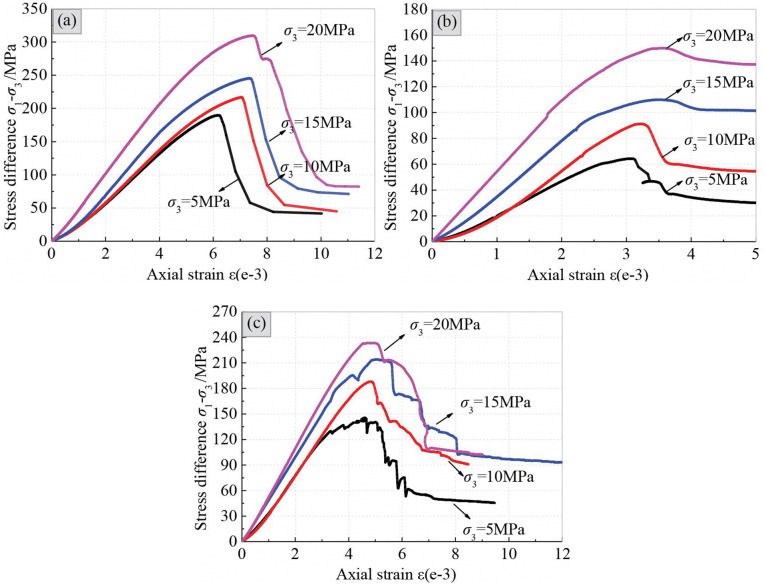
Stress-strain curves of specimens subjected to triaxial compression, (a) *α* = 90°, (b) *α* = 30° and (c) *α* = 0°.

The stress-strain curves of specimens subjected to triaxial compression are shown in Fig 6. Under the initial differential stress, the compaction phenomenon of specimens is not as obvious as that in the absence of lateral constraint. This variation can be attributed to the fact that the preexisting defects have already been compression-closed to a certain extent in the process of applying the confining pressure. Overall, the peak stress and slope of the linear elastic segment of the stress-strain curves gradually increase with increasing confining pressure, indicating that the confining pressure exerts a significant effect on enhancing the strength and deformation resistance of schist. In the state of lateral constraint, the post peak stress will drop to a residual value, which then remains stable with increasing strain. It suggests that even in the event of failure, the specimens with lateral constraint still have residual strength to exert a certain load-bearing capacity. Moreover, the ratios of residual strength to peak strength (Table 1) in different loading directions follow the order of *α* = 30° (0.50–0.93)> *α* = 0° (0.34–0.54)> *α* = 90° (0.23–0.34). Overall, the axial strains corresponding to the peak points tend to increase as the confining pressure increases.

**Table 1:** Mechanical parameter values of specimens subjected to triaxial compression.

Confining pressure (MPa)	Parameter values of specimens at different orientation angles								
	*α* = 90°			*α* = 30°			*α* = 0°		
	Peak strength (MPa)	Residual strength (MPa)	Elastic modulus (GPa)	Peak strength (MPa)	Residual strength (MPa)	Elastic modulus (GPa)	Peak strength (MPa)	Residual strength (MPa)	Elastic modulus (GPa)
5	194.44	44.30	36.30	69.22	34.66	27.32	150.34	50.50	44.83
10	226.57	52.50	38.47	101.10	64.64	35.28	197.97	101.04	48.01
15	260.12	88.80	45.52	124.93	116.00	36.95	229.13	124.36	50.62
20	329.38	100.30	52.50	169.83	157.20	38.06	253.22	130.85	56.38

As in uniaxial compression, the stress-strain curves of the specimens with *α* = 90° still show obvious strain softening characteristics within the applied confining pressure range. However, it was found that as the confining pressure increases, the yield stage of specimens gradually prolongs, accompanied by a corresponding increase in the amount of plastic deformation. In addition, the descent slope of post peak stress also decreases under higher confining pressure. These findings suggest that the increased confining pressure leads to a certain degree of reduction in brittleness of specimens with *α* = 90°.

Although the strain softening phenomenon still exists in the specimens with *α* = 30°, the slope and the magnitude of the post-peak stress drop significantly decrease with the increase of confining pressure. Signs of a yield plateau appear in the stress-strain curve under higher confining pressure, exhibiting a tendency of ideal plasticity. Obviously, as the confining pressure increases, the specimen with *α* = 30° gradually transitions from brittleness to ductility. The critical confining pressure of brittle-ductile transition can be estimated by fitting the relationship between confining pressure and the differentials of peak and residual stress. The estimated results show that the critical confining pressure is 28.3 MPa, close to 0.6 times the uniaxial compressive strength.

At the confining pressure of 5 MPa, the stress-strain curve for the specimen with *α* = 0° exhibits frequent fluctuations, similar to the phenomenon in uniaxial compression. As the confining pressure increases, the fluctuations gradually weaken or even disappear, and the slope of the post-peak stress drop also tends to decrease. At the confining pressure of 15 MPa and 20 MPa, the post-peak curves contain several spaced stress platforms, showing a stepwise drop phenomenon. It suggests that the specimens undergo plastic damage.

#### 3.2.2 Strength anisotropy.

**Fig 7:**
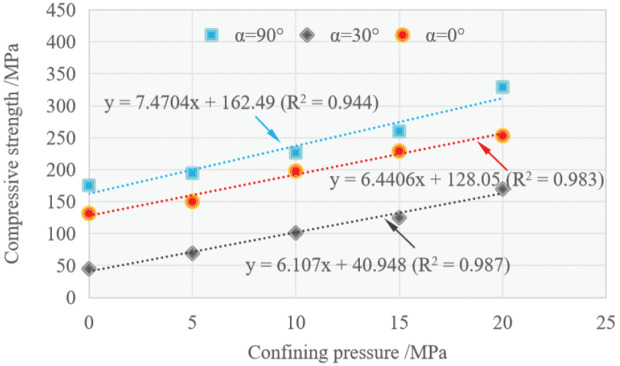
Variation of compressive strength with confining pressure.

Fig 7 presents the uniaxial and triaxial compressive strengths to illustrate their relationship with confining pressure and orientation angle. It clearly demonstrates that within the applied confining pressure range, the compressive strength of specimen with *α* = 90° is always larger than that with *α* = 30° and *α* = 0°, and the minimum strength of schist is consistently at *α* = 30°.

The enhancement effect of confining pressure on strength is clearly revealed in the scatter plot of compressive strength versus confining pressure for specimens with different orientation angles (Fig 7). The data can be adequately fitted by straight lines. Therefore, the strength of the specimen varying with the confining pressure can be well characterized by the Mohr-Coulomb criterion. The relevant equations are applied to quantitatively determine the strength parameters of schist at *α* = 90°, 30° and 0°. The expression of principal stress for Mohr-Coulomb criterion is:


$$\sigma_{\text{1}}=\;\frac{1+sin\phi }{1-sin\phi }\sigma_{\text{3}}\text{+}\frac{2ccos\phi }{1-sin\phi }\;$$ (1)

where ϕ and c represent the internal friction angle and cohesion, respectively.

Based on the results of linear fitting on the data, ϕ (°) and c (MPa) of the tested schist at *α* = 90°, 0° and 30° were respectively determined as follows: ϕ=49.81, c=29.72; ϕ=46.99, c=25.23; ϕ=45.94, c=8.28. By comparison, the maximum values of ϕ and c occur at *α* = 90°, while the minimum is found at *α* = 30°. Especially, the cohesion at *α* = 30° decreases significantly compared to that at *α* = 90° and 0°. Previous studies have revealed that within a certain range of confining pressure, anisotropic rocks subjected to compression generally exhibit macroscopic failure characteristics of sliding failure along weak planes at *α* = 30° [13,17]. In the present study, it is assumed that the schist specimen with *α* = 30° slips thoroughly along the schistosity planes within the applied confining pressure range. Then, the following expression of deviatoric stress proposed by Jaeger [33] for sliding along the discontinuity was employed:


$$\sigma_{1(\beta )}\text{-}\sigma_{\text{3}}=\frac{2(c\text{w}+\sigma_{\text{3}}tan\phi \text{w})}{(1-tan\phi \text{w}cot\beta )sin2\beta }\;$$ (2)

where cw and ϕw are the cohesive force and internal friction angle of the weak planes. $\sigma_{1\left(\beta \right)}\;$ represents the maximum principal stress required to cause slip along the weak planes inclined at an angle *β*. The normal stress and shear stress on the weak plane inclined at an angle *β* are respectively:


$$\sigma_{\text{n}}=\frac{\text{1}}{\text{2}}\text{(}\sigma_{1\left(\beta \right)}+\sigma_{\text{3}}\text{)}+\frac{\text{1}}{\text{2}}\text{(}\sigma_{1\left(\beta \right)}-\sigma_{\text{3}}\text{)cos}2\beta \;$$ (3)

$$\tau =\frac{\text{1}}{\text{2}}\text{(}\sigma_{1\left(\beta \right)}-\sigma_{\text{3}}\text{)}sin2\beta$$ (4)

**Fig 8:**
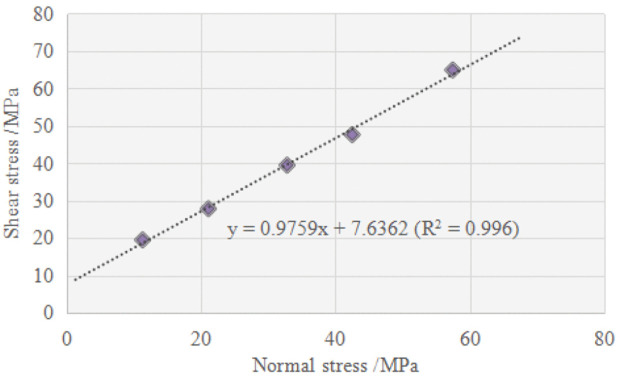
Variation of shear stress with normal stress on the sliding failure plane.

According to the assumption, the inclination angle of weak plane in case of sliding failure was taken as *β* = 90°- *α* = 60°. The confining pressure and corresponding compressive strength of the specimens with *α* = 30° were substituted into the Eqs. (3) and (4) to obtain several sets of (τ, $\sigma_{\text{n}}$), which are plotted in Fig 8. It shows that these data can be linearly fitted well, and the strength parameters were determined to be ϕw=44.30° and cw=7.64 MPa. The set of values is quite close to the parameter values obtained from Eq. (1). This close match therefore proves the validity of assumption that within the applied confining pressure range, the tested schist always undergoes sliding failure along the schistosity planes at *α* = 30°. Furthermore, by averaging these two sets of values, the strength parameters of weak planes were accurately determined to be ϕw=45.12° and cw=7.96 MPa, which are crucial for evaluating the stability of surrounding rocks with weak schistosity planes in the project area.

**Fig 9:**
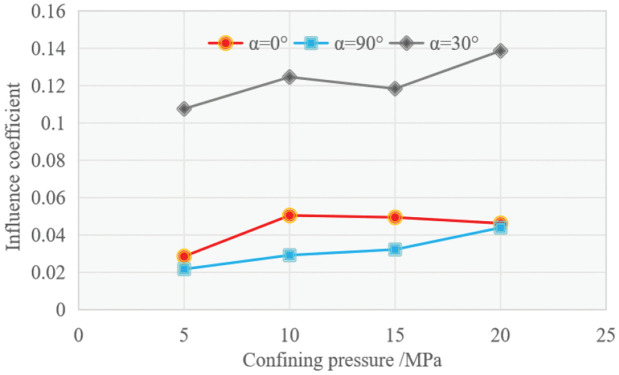
Comparison of influence coefficients at different orientation angles.

In order to visually compare the enhancement effects of confining pressure on the compressive strength of schist at *α* = 90°, 30° and 0°, a quantitative indicator, namely the influence coefficient δ of confining pressure on compressive strength, was proposed, which is expressed as follows:


$$\delta_{\left(\alpha \right)}=\frac{\sigma_{\text{c}\left(\alpha \right)}-\sigma_{\text{uc}\left(\alpha \right)}}{\sigma_{\text{uc}\left(\alpha \right)}*\sigma \text{3}}$$ (5)

where $\sigma_{\text{uc}\left(\alpha \right)}$ and $\sigma_{\text{c}\left(\alpha \right)}$ are the compressive strengths under uniaxial and triaxial (at confining pressure $\sigma_{\text{3}}$) loading, respectively, both measured at an orientation angle *α.*. The variations of δ as a function of $\sigma_{\text{3}}$ for *α* = 90°, 30° and 0° are shown in Fig 9. The results show that δ at *α* = 30° is 0.11**–**0.14, significantly larger than that at *α* = 90° (0.02**–**0.04) and *α* = 0° (0.03**–**0.05). It reveals that the confining pressure has a most significant effect on the compressive strength of rock at *α* = 30°, while the response of strength to confining pressure is least sensitive for rock at *α* = 90°.

The applied confining pressure causes the existing open defects in the rock to close, making the structure of rock denser, thereby enhancing the rock's ability to resist failure. The difference in the enhancement effect of confining pressure on the strength of schist at different orientation angles is likely related to the role played by directional cracks in the failure process of rock, which will be elaborated in the following text. It is the orientation dependence of the confining pressure effect that yields the systematic response of the strength anisotropy to confining pressure for the schist.

Generally, the ratio $\sigma_{min}/\sigma_{max}$ of maximum (at *α* = 90°) to minimum (at *α* = 30°) compressive strength is applied to evaluate the strength anisotropy degree of rock. When the confining pressure varies from 0 MPa to 20 MPa, the corresponding $\sigma_{min}/\sigma_{max}$ is 3.90, 2.81, 2.24, 2.08, and 1.94, respectively. This trend reveals that the strength anisotropy of schist is gradually weakened by the increasing confining pressure. The conclusion is consistent with the previous findings [15,31,34,35].

**Fig 10:**
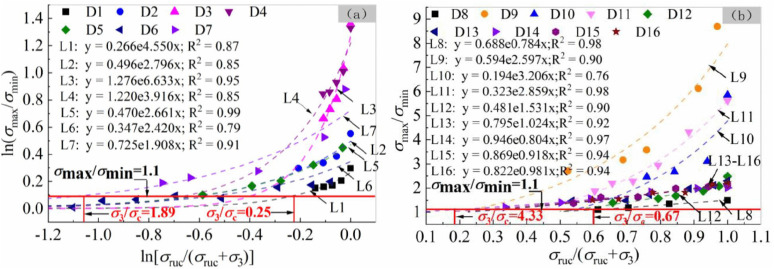
Effect of confining pressure on strength anisotropy for rocks with weak planes.

In order to further explore the general pattern of how confining pressure influences strength anisotropy of rocks with inherent weak planes, some test results of predecessors are statistically analyzed [17,31,36–46], and plotted in the coordinate system of $\sigma_{max}/\sigma_{min}$ versus $\sigma_{\text{ruc}}/\;\left(\sigma_{\text{ruc}}+\sigma_{\text{3}}\right)$ or $ln(\sigma_{max}/\sigma_{min}\text{)}$ versus $\begin{array}{c} ln\left[\sigma_{\text{ruc}}/\;\left(\sigma_{\text{ruc}}+\sigma_{\text{3}}\right)\right] \end{array}$ (Fig 10). It should be pointed out that the accuracy of the data fitting was taken as the most important consideration to select the appropriate coordinate system.

Although the data collected from previous studies were statistically analyzed in two different coordinate systems, a good functional relationship always exists between the horizontal and vertical coordinates for each group of data. In logarithmic and non-logarithmic coordinate systems, the relationships are respectively described as follows:


$$\text{y}=a^{*}exp\left(bx\right)$$ (6)

$$\text{y'}=c^{*}exp(dx\text{'})$$ (7)

where y and y’ represent $ln(\sigma_{max}/\sigma_{min}\text{)}$ and $\sigma_{max}/\sigma_{min}$, respectively; x and x’ represent $\begin{array}{c} ln\left[\sigma_{\text{ruc}}/\;\left(\sigma_{\text{ruc}}+\sigma_{\text{3}}\right)\right] \end{array}$ and $\sigma_{\text{ruc}}/\;\left(\sigma_{\text{ruc}}+\sigma_{\text{3}}\right)$, respectively. The fitted parameters, namely *a*, *b*, *c*, and *d*, have their respective indicative meanings.

The parameter value *a* is approximately equal to the natural logarithm of strength anisotropy degree under uniaxial compression. A larger *a* value indicates more significant anisotropy in the unconfined rock, suggesting that the weak planes have a more pronounced effect on uniaxial compressive strength. The parameter *b* characterizes the sensitivity of the anisotropy degree to changes in confining pressure in the logarithmic coordinate system. A larger *b* value indicates that an increase in confining pressure more significantly reduces the rock anisotropy. The parameter *c* is a fitting constant term. Its physical meaning is the initial scaling factor of the strength anisotropy degree when confining pressure is zero, which is related to the composition, structure, and weak plane development characteristics of rocks. The parameter *d* characterizes the sensitivity of the anisotropy degree to changes in confining pressure in the non-logarithmic coordinate system. A larger *d* value illustrates the significant regulatory effect of confining pressure on rock anisotropy. Exp (*d*) * *c* is entirely dependent on the strength anisotropy degree of uniaxially compressed rocks.

The uniform functional form implies a consistent weakening law of the confining pressure on the anisotropy of rocks with weak planes. It can be predicted that at a certain critical confining pressure, the anisotropic rock will tend toward an isotropic state, and the effect of weak planes on the mechanical properties of rock will be quite weak. The significance of critical confining pressure lies in defining the stress threshold that distinguishes anisotropic behavior from behavior that can be simplified as isotropic, thus providing quantitative basis for engineering design and analysis. Based on this threshold, scientific decision-making support can be provided in the simplification of numerical simulations, selection of support schemes, optimization of construction techniques, and risk assessment of geological hazards.

The fitted exponential function is competent for predicting the critical condition under which rocks behavior transitions from anisotropic to isotropic. According to the classification of strength anisotropy [46], rocks with strength anisotropy degrees of 1.0**–**1.1 are considered isotropic. The statistical results indicate that when $\sigma_{max}/\sigma_{min}$ is equal to 1.1, the ratio $\sigma_{\text{3c}}/\sigma_{\text{ruc}}$ of critical confining pressure to the most representative uniaxial compressive strength ranges from 0.25 to 4.33 for various types of rocks (Fig 10). For most data sets, the predicted critical ratio is less than 1, while for a considerable portion of data sets, the ratio is greater than 1. This differs from the research findings of Ramamurthy et al. [31], who believed that the critical ratio does not exceed 1. Tien et al. [13] fabricated layered synthetic-rock samples with a $\sigma_{\text{ruc}}$ of 66 MPa in the laboratory. They predicted that when the confining pressure reached 120 MPa, the failure of anisotropic samples was independent of discontinuous weak planes, suggesting that the mechanical response of samples transitioned from anisotropic to isotropic at $\sigma_{\text{3c}}/\sigma_{\text{ruc}}$=1.82. In the present study, the $\sigma_{\text{3c}}/\sigma_{\text{ruc}}$ of most rocks ranges from 0.50 to 3.00, which combines the results from Tien et al. [13] and Ramamurthy et al. [31].

**Fig 11:**
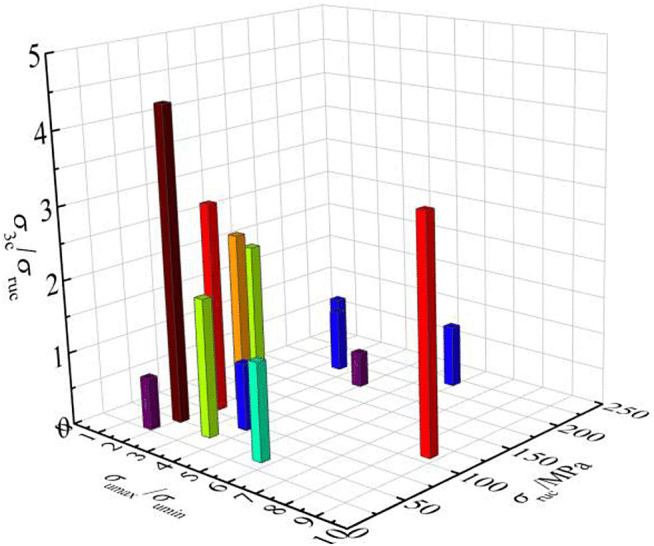
Response of critical ratio ($\sigma_{\text{3c}}/\sigma_{\text{ruc}}$) to the most representative uniaxial compressive strength ($\sigma_{\text{ruc}}$) and strength anisotropy degree under uniaxial compression ($\sigma_{\text{u}max}/\sigma_{\text{u}min}$).

The correlation between critical ratio $\sigma_{\text{3c}}/\sigma_{\text{ruc}}$ and most representative uniaxial compressive strength $\sigma_{\text{ruc}}$ as well as strength anisotropy degree under uniaxial compression $\sigma_{\text{u}max}/\sigma_{\text{u}min}$ was attempted to explore. A qualitative relationship can be clearly revealed from Fig 11, which is plotted based on previous experimental data. It shows that $\sigma_{\text{3c}}/\sigma_{\text{ruc}}$ has a negative correlation with $\sigma_{\text{ruc}}$ and $\sigma_{\text{u}max}/\sigma_{\text{u}min}$.

Then, multiple regression analysis was adopted to verify the relationship. Negative regression coefficients were obtained for the two independent variables, namely $\sigma_{\text{ruc}}$ and $\sigma_{\text{u}max}/\sigma_{\text{u}min}$, confirming the above qualitative conclusion. It means that the larger most representative uniaxial compressive strength and strength anisotropy degree under uniaxial compression, the smaller the critical ratio will be. Due to insufficient data, the specific functional relationship among the $\sigma_{\text{3c}}/\sigma_{\text{ruc}}$, $\sigma_{\text{ruc}}$ and $\sigma_{\text{u}max}/\sigma_{\text{u}min}$ is still unclear, which needs to be further explored in the future. The statistical results show that, depending on the type of rock, the critical confining pressure $\sigma_{\text{3c}}$ can be as low as 30 MPa or as high as over 300 MPa. Overall, the $\sigma_{\text{3c}}$ of metamorphic rocks with gneissosity or schistosity planes is generally higher than that of sedimentary rocks with bedding planes. For sedimentary rocks, the $\sigma_{\text{3c}}$ of sandstone is significantly lower than that of shale. It is likely that the $\sigma_{\text{3c}}$ closely depends on the development degree of weak planes. Rocks with more developed weak planes have higher $\sigma_{\text{3c}}$.

#### 3.2.3 Failure mode.

(a) *α=0°*

**Fig 12:**
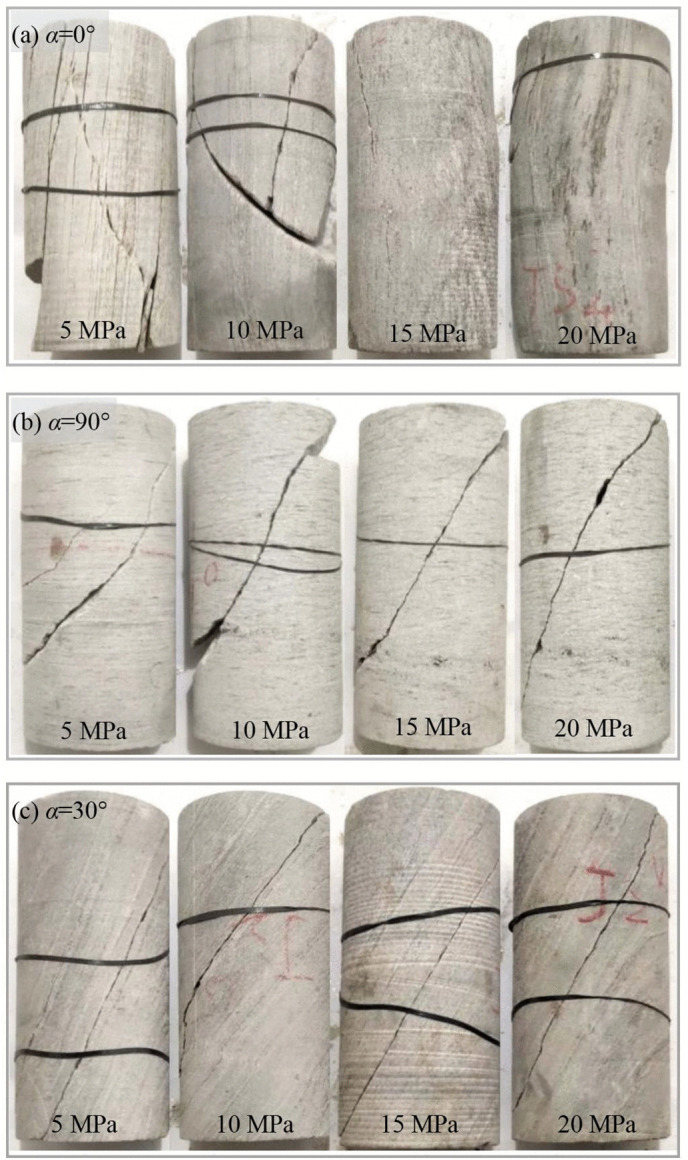
Failed schist specimens subjected to triaxial compression.

At *α* = 0°, the failure mode of the tested schist changes significantly with the variation of the confining pressure. The specimen subjected to the confining pressure of 5 MPa undergoes a splitting and shear composite failure along and across the schistosity planes (Fig 12a), similar to the test results of Zhou et al. [39]. Under tensile stress from lateral deformation, multiple splitting fractures progressively develop along the schistosity planes during loading, resulting in frequent fluctuations in the stress-strain curve. These splitting fractures are connected by shear planes diagonally intersecting with the axis of specimen.

**Fig 13:**
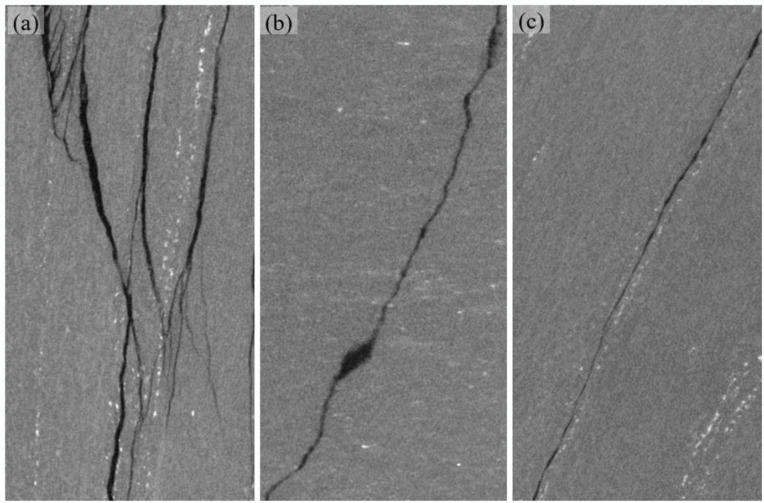
CT scanning images, (a) *α* = 0° at the confining pressure of 5 MPa, (b) *α=*  90° at the confining pressure of 10 MPa and (c) *α* = 30° at the confining pressure of 5 MPa. The vertical direction in each image corresponds to the specimen axial direction.

The content and aggregation degree of mica have a significant impact on the failure mode of rock. Under the same confining pressure, the similar rock with relatively low content and aggregation degree of mica only exhibits shear failure in absence of multiple splitting fracture planes, which is different from the failure mode of rock in the current study. The tested schist has high content and aggregation degree of mica, contributing to the more developed schistosity planes. As a result, the rock is prone to being induced tensile fracture planes under the condition of low confining pressure. Following the triaxial compression test, a high-precision industrial computed tomography (CT) scanning system with a resolution of nearly 25 um was employed to examine the key deformation and failure zones of the damaged specimens. This was done to clearly detect the distribution of internal cracks. As shown in Fig 13a, under a relatively low confining pressure of 5 MPa, the specimen was subjected to a combined tensile-shear failure, predominantly characterized by splitting along the schistosity planes.

**Fig 14:**
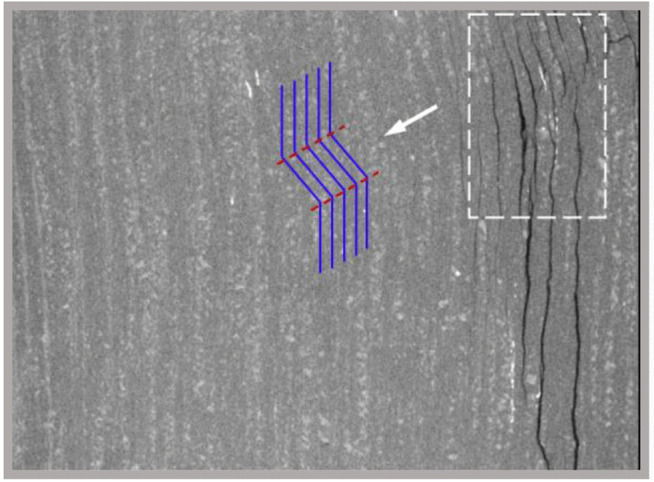
Localized kinking revealed by CT scanning.

When the confining pressure increases to 10 MPa, the number of tensile fracture planes is greatly reduced, and the shear fracture plane exists as the primary failure plane. At the confining pressure of 15 MPa, the number of failure planes in the specimen further decreases, and the tension and shear mechanisms are included in the same fracture plane. In addition, bulging of the specimen towards one side was observed, indicating the occurrence of plastic deformation dominated by localized kinking, as confirmed by CT scanning (Fig 14). At the confining pressure of 20 MPa, no throughgoing fracture plane exists in the specimen, and the bulging caused by kinking becomes more obvious (Fig 12a), leading to the increase of tensile stress on the bulging side. As a result, local fracture planes along the weak planes arise.

It can be seen that under relatively high confining pressure, the mica-rich specimen slowly adjusts the stress change in a form of plastic deformation under compression parallel to the schistosity planes. Therefore, the stress-strain curve slowly drops in a stepwise from after the peak point, which differs from the behavior of the similar specimen containing less abundant mica. Under the same confining pressure, brittle failure occurs in the form of throughgoing shear fractures due to the higher proportion of strong mineral phase in the low-mica specimen. Correspondingly, a rapid and continuous drop appears in the post peak section of the stress-strain curve.

(b) *α=90°*

At *α* = 90°, the specimen subjected to a relatively low confining pressure of 5 MPa undergoes a tensile and shear composite failure with a primary fracture plane across the schistosity planes (Fig 12b). In the middle part of the specimen, the fracture plane exhibits discernible sub-axial development, indicating that the cracks induced by tensile stress are capable of overcoming the low lateral constraints to expands. Towards the end surfaces of the cylinder specimen, the fracture plane develops obliquely to the specimen axis. This can be attributed to the suppression of tensile cracks by enhanced lateral constraints stemming from a certain degree of friction on the end surfaces. The overall shape of the fracture plane is quite irregular due to its significant turning in the extending direction. In addition, local secondary fracture planes are present in the specimen at the confining pressure of 5 MPa.

When the confining pressure increases to 10 MPa, a primary fracture plane that intersects diagonally with the axial stress develops through the entire specimen. Compared with the failure morphology at the confining pressure of 5 MPa, no axially developed fracture zones are visible on the specimen surface. However, localized tensile fractures are still clearly discernible in the CT image (Fig 13b). These observations indicates that the increased confining pressure weakens the tensile mechanism and enhances the shear mechanism during the process of rock failure. In the middle part of the specimen, the fracture plane tends to expand at a small angle to the specimen axis (Fig 12b). Approaching the end surfaces of specimen, the extending direction of fracture plane changes at a larger angle to the specimen axis due to the friction effect. At the turning point of the extending direction of failure plane, a nearly horizontal fracture plane appears, which cuts out a local block together with the primary failure plane.

With increasing confining pressure, the geometry of the shear failure plane evolves toward a more regular configuration, while the development of secondary fracture planes is suppressed. However, the characteristic of a certain degree of turning in the extending direction of the failure plane is still retained. The increase in confining pressure enhances inter-granular friction resistance, as evidenced by the decrease in the angle *θ* (*θ* represents the angle between the failure plane in the central region of the specimen and the specimen axis) from about 45° at 5 MPa to approximately 30° at 20 MPa.

In addition, at *α* = 90°, a large amount of crystalline debris remains on the fracture surfaces, indicating the appearance of considerable transgranular cracks during the failure process of specimen. These cracks not only cut off flaky mica clusters, but also penetrate through many granular minerals. Apart from transgranular cracks, intergranular cracks along the boundaries of minerals and cleavage cracks in randomly oriented mica arise in the specimen. The diversity of crack types and the differences in local extending directions of various cracks lead to rough and uneven fracture surfaces in the specimen with *α* = 90° [21].

(c) *α=30°*

At *α* = 30°, the failure mode of the tested schist appears to be independent of the confining pressure. A relatively flat failure plane develops along the schistosity plane (Figs 12c and 13c), which is attributed to the crack propagation and connection along the cleavage in the aggregated mica clusters or directional cracks at the boundaries of mica. Under low confining pressure, the failure plane in the tested schist differs from that in schist with low content and aggregation degree of mica. The latter is generally characterized by relatively rough, even stepped failure surfaces.

Previous studies have shown that the foliated rocks contain many intermittently distributed and parallel weak layers composed of multiple flaky mineral clusters, and there exists more or less strong mineral phase as “rock bridge” between the mineral clusters in the same weak layer [17]. When the quartz mica schist is subjected to compressive load, the concentration of tensile and shear stresses is prone to forming at the tips of mica. The stress concentration facilitates the slip deformation along the cleavage plane of the flaky mineral, which in turn induces tensile and shear cracks in the zone of strong phase adjacent to mica grains [47]. Under the favorable condition of low confining pressure, tensile cracks tend to propagate toward the specimen axis [26].

For the case of low aggregation degree of mica, the strong phase between the mica clusters in the same layer accounts for a large proportion. The mica clusters in the neighboring weak layers will be connected by the axially developed cracks, eventually leading to a relatively rough failure plane. For the case of high aggregation degree of mica, the strong phase between the mica clusters in the same layer accounts for a small proportion. The shear cracks initiated following the appearance of tensile cracks will precede axially developed cracks to connect the mica clusters in the same weak layer, eventually resulting in a relatively flat failure plane.

In mica clusters, cracks propagate along cleavage planes or existing directional cracks. Previous studies have shown that the stress at the tips of slender cracks can be several times higher than the applied stress, and stress concentrations increase with the length of existing defects [48]. Therefore, those directional large-scale cracks play an important role in the progressive failure of specimens with *α* = 30°. In addition, the degree of stress concentration also depends on the orientation angle of flaky minerals and existing defects. Maximum stress concentration is generally at the orientation angle of 30°–45° [49], accounting for the ease of failure in schist at *α* = 30°. Owing to the mechanical weakness of the cleavage plane and existing defects, the tested schist containing high content and aggregation degree of mica possess quite low strength at *α* = 30° relative to that at *α* = 90°, resulting in a considerable anisotropy degree for the rock.

Although the geometry of failure plane is independent of the confining pressure, its straightness is reduced under the high confining pressure of 20 MPa. It is attributed to the fact that the specimens sheared along the weak planes with some development of “kinks” [31].

**Fig 15:**
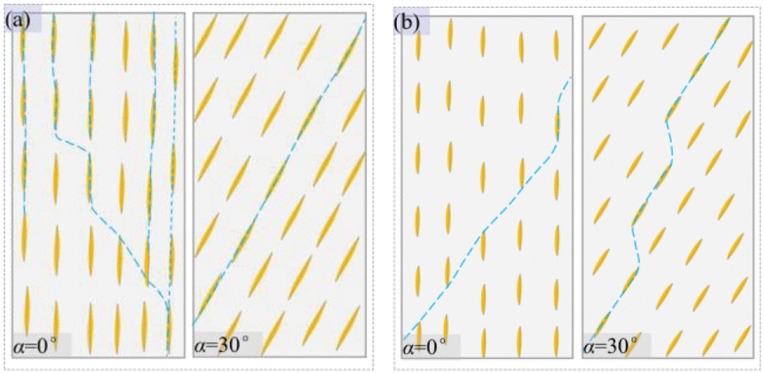
Diagrammatic drawing of failure modes of schist under the condition of low confining pressure, (a) schist containing high content and aggregation degree of mica and (b) schist containing low content and aggregation degree of mica. In the drawing, represents the flaky mica cluster and represents a weak layer including multiple mica clusters. represents the strong mineral phase composed of granular minerals.

In summary, confining pressure has a significant impact on the progressive failure of the tested schist. Specifically, the tensile failure mechanism is suppressed and plastic deformation is strengthened by the increasing confining pressure, and high confining pressure even cause kinking due to the presence of abundant flaky minerals. Apart from the confining pressure, the failure of schist is highly dependent on the loading direction. The schist undergoes structural instability along the mechanical weak planes at *α* = 30°, while material failure mostly associated with mineral properties *α* = 90° and 0°. Therefore, the strength of specimen with *α* = 30° has a more sensitive response to changes in stress, accounting for its more significant enhancement effect of confining pressure. In addition, under relatively low confining pressure, the tested schist undergoes a splitting and shear composite failure at *α* = 0° and sliding shear failure at *α* = 30°. In contrast, the schist with low content and aggregation degree of mica suffers shear failure at *α* = 0° and sliding tension and shear composite *α* = 30° of (Fig 15).

## 4 Conclusions

In this study, the fabric characteristic and mechanical properties of schist containing high content and aggregation degree of mica were investigated using micro tests and uniaxial and triaxial compression tests. The main conclusions are as follows.

(1) The mica in the tested schist exhibits highly aggregated characteristics. The flaky minerals are aggregated and directionally arranged in a streamline pattern in the “matrix” composed of granular particles such as quartz, feldspar and calcite. There exist original defects in schist samples, including directional cracks developed along the cleavage in mica or the boundaries of flaky mineral and micropores randomly distributed at the particle gaps.
(2) All the specimens with different orientation angles undergo significant brittle failure under uniaxial compression. As functions of the orientation angle, the peak strength and elastic modulus of schist subjected to uniaxial compression vary in a shoulder form and U-shaped form, respectively.
(3) The confining pressure ranging from 5 MPa to 20 MPa has a linear enhancing effect on the compressive strength of schist specimens with the representative orientation angle. The influence coefficient δof confining pressure on compressive strength was proposed to quantitatively analyze the difference in response of enhancing effects to the loading direction. It was found that the confining pressure has a most significant effect on the strength of rock at *α* = 30°, while the response of strength to confining pressure is least sensitive for rock at *α* = 90°. As a result, the strength anisotropy degree of schist decreases regularly with increasing confining pressure. This understanding provides a direct basis for developing strength prediction models that account for confining pressure and structural plane orientation, serving as an important foundation for revising strength criteria of anisotropic rocks in deep engineering.
(4) The statistical analysis of the data from previous studies and the present study indicates that the relationship between the anisotropy degree of rocks with weak planes and confining pressure can be described by the exponential function. Mechanical behavior of rocks with weak planes will transition from anisotropic to isotropic at a certain critical confining pressure. The critical ratio $\sigma_{\text{3c}}/\sigma_{\text{ruc}}$ has a negative correlation with the most representative uniaxial compressive strength $\sigma_{\text{ruc}}$ and strength anisotropy degree under uniaxial compression $\sigma_{\text{u}max}/\sigma_{\text{u}min}$. This critical confining pressure can be used as an important criterion to evaluate whether an isotropic simplified model is applicable for the stability analysis of actual engineering rock masses under in-situ stress conditions, thereby optimizing the design process.
(5) Confining pressure and loading direction play significant roles in the failure mode of the schist. At *α* = 90°, the tensile and shear composite failure with relatively irregular fracture planes appears in the schist under the low confining pressure. The increase in confining pressure suppresses the fracture related to the tensile mechanism, leading to the appearance of pure shear failure plane. The tested schist subjected to triaxial compression undergoes sliding shear failure at *α* = 30°, exhibiting a flat failure plane along the same weak layer even under the low confining pressure. The straightness of failure plane is reduced at the high confining pressure of 20 MPa due to the kink developed in weak schistosity planes. At *α* = 0°, the failure of the tested rock transitions from splitting and shear composite to plastic kinking in response to the increasing confining pressure. Furthermore, the failure modes of the tested sample at *α* = 0° and 30° differ from that of schist containing low content and aggregation degree of mica under the low confining pressure. It is attributed to the strong dependence of failure modes on fabric characteristics in foliated rocks. The above understanding is helpful in anticipating potential failure mechanisms based on rock mass structure and stress state in engineering practice, providing a theoretical reference for formulating targeted prevention and control measures.

Future work should include replicate tests at critical mechanical states (e.g., the orientation angle corresponding to the minimum strength) to quantify the statistical variability. In addition, the proposed influence coefficient of confining pressure and critical confining pressure under in-situ engineering stress paths should be further validated and calibrated, and subsequently developed into applicable design criteria for assessing the strength and stability of rock masses in deep engineering.

## Supporting information

S1 File Relevant data. Experimental measurement and statistical analysis data.
(XLS)
